# The Effectiveness of Image Augmentation in Deep Learning Networks for Detecting COVID-19: A Geometric Transformation Perspective

**DOI:** 10.3389/fmed.2021.629134

**Published:** 2021-03-01

**Authors:** Mohamed Elgendi, Muhammad Umer Nasir, Qunfeng Tang, David Smith, John-Paul Grenier, Catherine Batte, Bradley Spieler, William Donald Leslie, Carlo Menon, Richard Ribbon Fletcher, Newton Howard, Rabab Ward, William Parker, Savvas Nicolaou

**Affiliations:** ^1^Rady Faculty of Health Sciences, University of Manitoba, Winnipeg, MB, Canada; ^2^School of Mechatronic Systems Engineering, Simon Fraser University, Burnaby, BC, Canada; ^3^Nuffield Department of Surgical Sciences, University of Oxford, Oxford, United Kingdom; ^4^School of Electrical and Computer Engineering, University of British Columbia, Vancouver, BC, Canada; ^5^Department of Emergency and Trauma Radiology, Vancouver General Hospital, Vancouver, BC, Canada; ^6^Department of Radiology, Louisiana State University Health Sciences Center, New Orleans, LA, United States; ^7^Department of Physics & Astronomy, Louisiana State University, Baton Rouge, LA, United States; ^8^Biomedical and Mobile Health Technology Laboratory, Department of Health Sciences and Technology, ETH Zurich, Zurich, Switzerland; ^9^D-Lab, Massachusetts Institute of Technology, Cambridge, MA, United States; ^10^Department of Radiology, Faculty of Medicine, University of British Columbia, Vancouver, BC, Canada

**Keywords:** radiology, corona virus, transfer learning, data augmentation, chest X-ray, digital health, machine learning, artificial intelligence

## Abstract

Chest X-ray imaging technology used for the early detection and screening of COVID-19 pneumonia is both accessible worldwide and affordable compared to other non-invasive technologies. Additionally, deep learning methods have recently shown remarkable results in detecting COVID-19 on chest X-rays, making it a promising screening technology for COVID-19. Deep learning relies on a large amount of data to avoid overfitting. While overfitting can result in perfect modeling on the original training dataset, on a new testing dataset it can fail to achieve high accuracy. In the image processing field, an image augmentation step (i.e., adding more training data) is often used to reduce overfitting on the training dataset, and improve prediction accuracy on the testing dataset. In this paper, we examined the impact of geometric augmentations as implemented in several recent publications for detecting COVID-19. We compared the performance of 17 deep learning algorithms with and without different geometric augmentations. We empirically examined the influence of augmentation with respect to detection accuracy, dataset diversity, augmentation methodology, and network size. Contrary to expectation, our results show that the removal of recently used geometrical augmentation steps actually improved the Matthews correlation coefficient (MCC) of 17 models. The MCC without augmentation (MCC = 0.51) outperformed four recent geometrical augmentations (MCC = 0.47 for Data Augmentation 1, MCC = 0.44 for Data Augmentation 2, MCC = 0.48 for Data Augmentation 3, and MCC = 0.49 for Data Augmentation 4). When we retrained a recently published deep learning without augmentation on the same dataset, the detection accuracy significantly increased, with a $\chi_{\text{McNema}{\text{r}}^{\text{′}}\text{s statistic}}^{2}=163.2$ and a *p*-value of 2.23 × 10^−37^. This is an interesting finding that may improve current deep learning algorithms using geometrical augmentations for detecting COVID-19. We also provide clinical perspectives on geometric augmentation to consider regarding the development of a robust COVID-19 X-ray-based detector.

## Introduction

More people are being infected with COVID-19 every day (1); therefore there is a need for a quick and reliable technology to help with the screening and management of the virus. Recent research (2) has shown that the combination of deep learning and chest X-rays could be faster and less expensive than the gold standard for COVID-19 diagnosis, which is a laboratory technique called reverse transcriptase polymerase chain reaction (PCR). It is therefore expected that this area of research will attract more researchers and that more papers will be published on this topic. Deep learning provides the ability to learn and nonlinearly associate high-dimensional features in X-ray images that feature COVID-19 (3). One of the techniques used during the training and testing phases is data augmentation, which is used to make the deep learning model more robust to different types of noise, as well as increase the training dataset, which is typically needed in clinical applications.

It should be noted that data augmentation is commonly used in binary classification in cases where a large imbalance exists between the size of the two classes being used in a machine learning model. Algorithms such as SMOTE (4) are often used to augment the minority class by intelligently synthesizing new data without overfitting. However, the use of data augmentation to improve the robustness and generalizability of the model has been less explored and is the primary motivation for this paper. There are two ways to apply data augmentation: (1) class-balancing oversampling (number of synthesized images more than in the training dataset), (2) replacement (number of synthesized images equals the number of images in the training dataset). The former is the most used data augmentation approach (5), which is being used to boost the number of images; however, to our knowledge, the latter is not discussed in the literature. Our focus here directly assesses the impact of data augmentation with replacement.

Recent studies (6–8) of COVID-19 detection from chest X-rays have used several data augmentation techniques to improve the testing accuracies of deep learning models, including random rotation, translation, and horizontal flipping. In some cases, two methods (translation and rotation) have been used at the same time. Researchers (9, 10) performed data augmentation with rotation and both horizontal and vertical flipping to increase the diversity of the data set, but it is not clear whether these steps were carried out randomly or at the same time. Another study (11) applied a data augmentation step to ensure model generalization that used only a random image rotation. In contrast, other studies (12–18) have attempted to apply deep learning without a data augmentation step, which has created controversy over the use of data augmentation for detecting COVID-19 specifically, as well as for detecting abnormalities in X-Ray images in general.

Note that none of these studies (6–17) gave a reason for including or not including an augmentation step. In other words, they did not compare the models with and without augmentation to support their claims of improvement. We therefore sought to examine the impact of the augmentation step on detecting COVID-19 using X-ray images by investigating the impact of data augmentation on an optimized deep neural network (19).

## Method

To test the efficacy of the augmentation step, we examined our most recently published deep learning method (19), DarkNet-19, with and without data augmentation. The analysis was carried out using MATLAB 2020a on a workstation (GPU NVIDIA GeForce RTX 2080Ti 11 GB, RAM 64 GB, and Intel Processor I9-9900K @3.6 GHz).

## Datasets

We created three datasets based on a publicly available dataset and two local datasets. The publicly available dataset is called “CoronaHack–Chest X-Ray-Dataset” (CHC-Xray; downloaded from https://www.kaggle.com/praveengovi/coronahack-chest-xraydataset). The first local dataset was collected from Vancouver General Hospital (VGH), British Columbia, Canada, and contains 58 COVID-19 X-ray images. More details about the CHC and VGH datasets are discussed in our recently published work (19). The second local dataset was collected by the Department of Radiology at Louisiana State University (LSU), USA, and contains 374 coincident CXR and PCR tests evaluated for 366 individual patients. The clinical characteristics of the 366 patients at the time of RT-PCR testing include: 178/366 male (49%) and 188/366 female (51%) patients, with a mean age of 52.7 years (range 17–98 years). Average patient body mass index (BMI) was 32.0 ± 9.7 *kg*/*m*^2^. More details about the LSU dataset are discussed in our recently published work (20). All X-ray images were used from the LSU dataset, no image was excluded.

The datasets used in the training and validation stages of our study are defined as follows:

- Dataset 1 was formed from CHC-Xray and consisted of 100 X-ray images (COVID = 50, healthy = 16, bacterial pneumonia = 16, non-COVID-19 viral pneumonia = 18).
- Dataset 2 was formed from LSU and consisted of 374 X-ray images (COVID = 198, non-COVID = 176)
- Dataset 3 was formed from CHC+LSU and consisted of 474 X-ray images (COVID = 248, non-COVID = 226)
- Dataset 4 was formed as a testing dataset based on a previous publication (19), which combined CHC-Xray and VGH datasets, with a total of 5,854 X-ray images (COVID = 58, healthy = 1,560, bacterial pneumonia = 2,761, non-COVID-19 viral pneumonia = 1,475). Note that Dataset 1 was used for training and validation during the development of the COVID-19 algorithm (19) and therefore it is used to retrain and revalidate the same algorithm without augmentation.

## Data Augmentation

The data augmentation steps examined in this study have been used in the literature for detecting COVID-19. Data augmentation procedures are intrinsically arbitrary and their justification is based upon empirical considerations (i.e., model performance) rather than fixed clinical considerations. Here, we will examine different data augmentation methods used recently in the literature. Our objective is to understand the impact of data augmentation and better understand whether one form of data augmentation is more useful than another. Four data augmentation methods proposed by Yoo et al. (6), Nishio et al. (21), Ahuja et al. (22), and Zhang et al. (23), implemented as follows:

- **Data Augmentation 1**: This augmentation step is proposed by Nishio et al. (21), which includes rotation within the range [−15, 15], translation in x- and y-axis within the range [−15, 15], horizontal flipping, scaling, and shear within the range 85–115%. The pseudo code for this augmentation method is as follows:
‘‘RandRotation,’’[-15 15],...
‘‘RandScale,’’[0.85 1.15],...
‘‘RandYReflection,’’true,...
‘‘RandXShear,’’[-**floor**(0.1*inputSize)
**floor**(0.1*inputSize)],... 
‘‘RandYShear,’’[-**floor**(0.1*inputSize)
**floor**(0.1*inputSize)],...
‘‘RandXTranslation,’’
[-**floor**(0.15*inputSize)
**floor**(0.15*inputSize)],...
‘‘RandYTranslation,’’
[-**floor**(0.15*inputSize)
**floor**(0.15*inputSize)]
- **Data Augmentation 2**: This augmentation step is proposed by Ahuja et al. (22), which includes shear operation within the range [−30, 30], random rotation within the range [−90, 90], and random translation from pixel range [−10, 10]. The pseudo code for this augmentation method is as follows:
‘‘RandRotation,’’[-90 90],...
‘‘RandXShear,’’[-30 30],...
‘‘RandYShear,’’[-30 30],...
‘‘RandXTranslation,’’[-10 10],...
‘‘RandYTranslation,’’[-10 10]
- **Data Augmentation 3**: This augmentation step is proposed by Zhang et al. (23), which includes random rotation (30-degree range) and horizontal flipping. The pseudo code for this augmentation method is as follows:
‘‘RandRotation,’’[-30 30],...
‘‘RandYReflection,’’true'
- **Data Augmentation 4**: This augmentation step is proposed by Yoo et al. (6), which includes random rotation by 10 degree, translation, and horizontally flipping. The pseudo code for this augmentation method is as follows:
‘‘RandRotation,’’[-10 10],...
 ‘‘RandYReflection,’’true',...
 ‘‘RandXTranslation,’’
[-**floor**(0.2*inputSize)
**floor**(0.2*inputSize)],... 
‘‘RandYTranslation,’’
[-**floor**(0.2*inputSize)
**floor**(0.2*inputSize)]

## Deep Learning Algorithms

We investigated 17 pretrained neural networks: AlexNet (24), SqueezeNet (25), GoogleNet (26), ResNet-50 (27), DarkNet-53 (28), DarkNet-19 (28), ShuffleNet (29), NasNet-Mobile (30), Xception (31), Place365-GoogLeNet (26), MobileNet-v2 (32), DenseNet-201 (33), ResNet-18 (27), Inception-ResNet-v28 (34), Inception-v3 (35), ResNet-101 (27), and VGG-19 (36). Each one of these pretrained neural networks has millions of parameters, and were originally trained to detect 1000 classes. Data augmentation and dropout were applied to all 17 networks, as part of the transfer learning that helps to combat overfitting (37, 38). This make these pretrained neural networks ideal for testing the efficacy of data augmentation. Dataset 1, Dataset 2, and Dataset 3 were used with cross validation *K* = 10, as it is based on experimental work (39) to reduce both bias and variance.

To use each pretrained network, the last fully-connected layer, also known as the last convolutional layer, is replaced with the number of filters equal to the number of classes. At this stage, we set the training parameters for the analysis using the same setting used a recently published work (19). We set the filter size to 1,1 and changed the number of filters to two based on the number of classes in the analysis (COVID-19 and Others). To ensure learning was faster in the new layer than in the transferred layers, we changed the learning rates by setting both “WeightLearnRateFactor” and “BiasLearnRateFactor” to 10. We set the solver to be “sgmd,” “InitialLearnRate” to 0.0001, ValidationFrequency to 5, and “MiniBatchSize” to 11. A recent study (40) showed that setting the “MaxEpochs” to 8 is sufficient for preventing overfitting, for reporting steady learning, and for generalizing a classifier.

## Evaluation Measures

To evaluate the performance of the 17 deep learning algorithm over the three datasets (Dataset 1, Dataset 2, and Dataset 3), accuracy and Matthews correlation coefficient (MCC) will be used. The accuracy is considered as the most popular adopted metrics in binary classification tasks; however, for imbalanced datasets, such as the case in our work, the MCC is recommended (41). Moreover, to confirm statical significance Wilcoxon rank sum is used.

To evaluate the performance of recently published model (19) with and without augmentation, McNemar's test will be used. Note that the recently published model (19) is referred to as Method I, while the same model without augmentation is refereed to as Method II in this work. The null hypothesis two models performs better than the other. Thus, we might consider the alternative hypothesis to be that the performances of the two models are not equal. However, in order to quantify and ensure that there is a significance between Method I and Method II, McNemar's test (42) is applied. Precisely we applied the corrected McNemar's test as recommended in (43), as follows:

(1) $$\begin{array}{l} \begin{array}{c} \chi^{2}=\frac{{\left(|B-C|-1\right)}^{2}}{B+C}, \end{array} \end{array}$$

where χ^2^ is the corrected McNemar's statistic, *B* is the number of X-ray images that were detected correctly by Method II and incorrectly detected by Method I, while *C* is the number of X-ray images that were detected correctly by Method I and incorrectly by Method II.

## Results and Discussion

**Figure 1:**
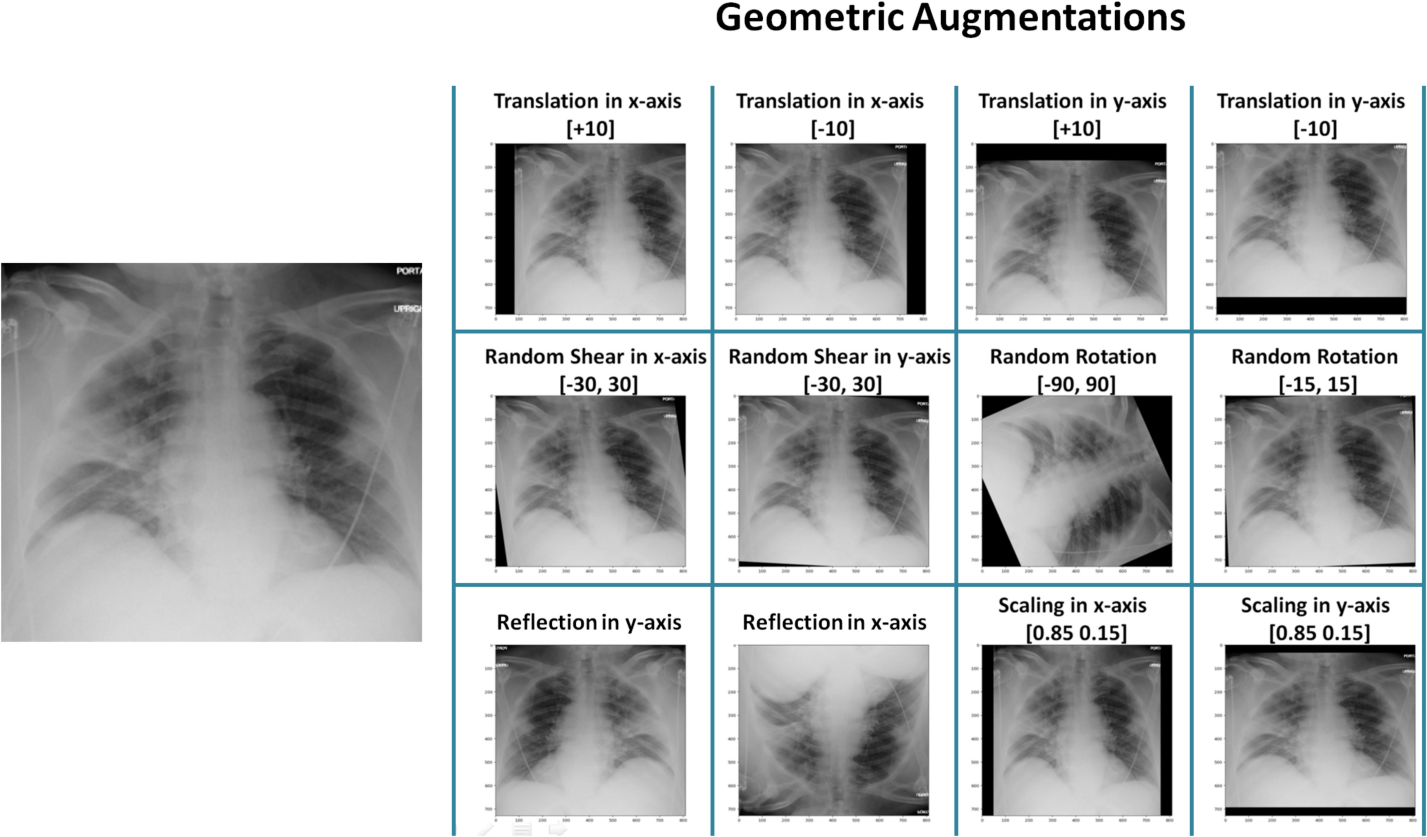
Application of different geometric augmentation transformations to a COVID-19 X-ray image.

Geometric augmentations are usually applied in combination to generate new augmented X-ray images. Figure 1 shows geometric augmentations that are applied individually to a COVID-19 X-ray image. This visualizes the impact of each geometric augmentation method and gives the reader an idea about their relevance. Figure 1 demonstrates 12 different augmentation methods. From left-right and top-down these are: translation in x-axis with +10 pixels, translation in x-axis with −10 pixels, translation in y-axis with +10 pixels, translation in y-axis with −10 pixels, random shear in x-axis within the range [−30,30], random shear in y-axis within the range [−30,30], random rotation within the range [−90,90], random rotation within the range [−15,15], horizontal reflection (or flipping), vertical reflection (or flipping), scaling in x-axis [0.85 1.15], and scaling in y-axis [0.85 1.15].

Table 1 shows the accuracy results of the four augmentation methods described above and the accuracy without applying the augmentation over three datasets. For simplicity, each geometrical augmentation transformation is presented in a separate column, as suggested in (44). The results show that data augmentation is not a required step and actually harmed the deep learning model in this case, by exposing it to a large amount of distorted (or noise) images. All augmentation methods scored lower validation accuracy than without augmentation. In other words, the augmenter trained the network on rotated and translated X-ray images that do not exist in a real-world scenario. To date, and to our current clinical knowledge, radiographic opacities associated with COVID-19 do not have a particular shape, size, or location. This in effect means geometric augmentation provides no added benefit, and can serve to reduce validation accuracy as shown here.

**Table 1:** Comparison of augmentation steps using three datasets.

						**Matthews correlation coefficient average (standard deviation)**			
	**Reflection**	**Scaling**	**Shearing**	**Translation**	**Rotation**	**Dataset 1** ** CHC**	**Dataset 2** **LSU**	**Dataset 3** **LSU+CHC**	**Average** **(standard deviation)**
None	–	–	–	–	–	0.93 (0.05)	0.24 (0.06)	0.37 (0.05)	0.51 (0.37)
Data Augmentation 1 (21)	✓	✓	✓	✓	✓	0.87 (0.1)	0.19 (0.06)	0.35 (0.04)	0.47 (0.35)
Data Augmentation 2 (22)	–	–	✓	✓	✓	0.8 (0.15)	0.2 (0.06)	0.33 (0.05)	0.44 (0.32)
Data Augmentation 3 (23)	✓	–	–	–	✓	0.87 (0.09)	0.21 (0.04)	0.34 (0.07)	0.48 (0.35)
Data Augmentation 4 (6)	✓	–	–	✓	✓	0.91 (0.05)	0.21 (0.04)	0.34 (0.06)	0.49 (0.37)

*The Matthews correlation coefficient (MCC) is used as an evaluation measure. An MCC coefficient of +1 represents a perfect prediction while −1 indicates total disagreement between prediction and observation. The average of 17 MCCs obtained from 17 deep learning algorithms with cross validation K = 10 is reported*.

Data Augmentation 4 outperformed the other three augmentation methods on three datasets, with an overall MCC = 0.49, suggesting that rotation, translation, and flipping could be used. On the contrary, Data Augmentation 2 scored the lowest validation MCC on the three datasets, with an overall MCC = 0.44, suggesting that a combination of rotation, translation, and shear are “not recommended” as an augmentation step during the process of developing a COVID-19 detector. We will focus on analyzing the behavior of Data Augmentation 2 and Data Augmentation 4; the former is worst, and the latter is the best relative to the four augmentation methods tested. Note that Data Augmentation 4, which scored the highest validation MCC compared to the other augmentation methods, did not outperform without augmentation.

**Figure 2:**
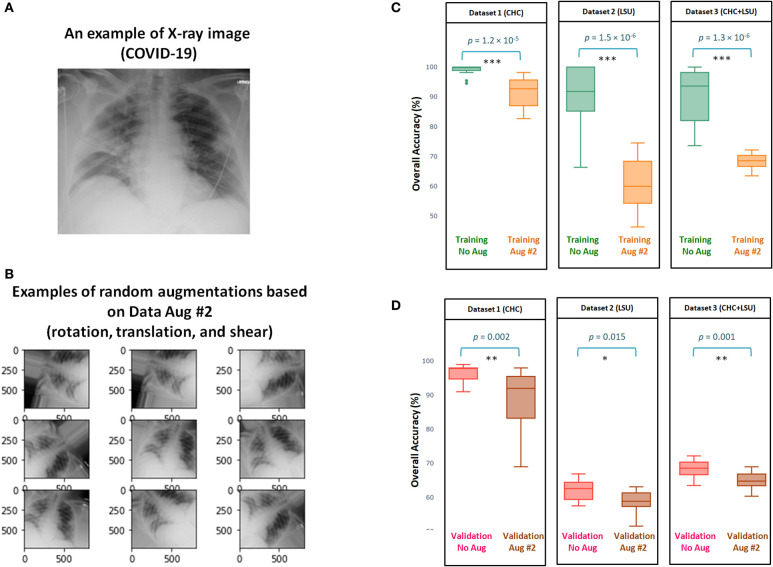
Efficacy of Data Augmentation 2 (22) on X-ray images with and without COVID-19. **(A)** An example of applying Data Augmentation 2, which includes shear operation within the range [−30, 30], random rotation within the range [−90, 90], and random translation from pixel range [−10, 10] to a COVID-19 X-ray image. **(B)** Comparing the overall accuracy of 17 deep neural networks to examine the efficacy of Data Augmentation 2 (21) when applied to three datasets. **(C)** Boxplots to show the overall statical different between training with and without augmentation over three datasets. **(D)** Boxplots to show the overall statical different between validation with and without augmentation. Note that results shown in **(C,D)** are obtained from cross validation with *K* = 10.

Figure 2A shows and example of X-ray image for a subject diagnosed with COVID-19 using PCR. Examples of random outputs for applying Augmentation 2 to Figure 2A are shown in Figure 2B. The overall training accuracy with Augmentation 2 is significantly lower (*p* << 0.0001 in all datasets) than the training accuracy without any augmentation method over three datasets, as shown in Figure 2C. However, the same finding was observed over the validation accuracy, with significance (*p* = 0.002, *p* = 0.015, *p* = 0.001, for Dataset 1, Dataset 2, Dataset 3, respectively), as shown in Figure 2D. Over three datasets, it is clear that Augmentation 2 helps prevent overfitting, however, it does not help with generalization compared to models without augmentation.

Interestingly, the overall validation accuracy without augmentation was significantly higher than the overall validation accuracy with Augmentation 2, with *p* < 0.05 over the three datasets, as shown in Figure 2D. This finding contradicts results usually reported (40) in computer vision where the overall validation accuracy without augmentation is lower than the overall validation accuracy with any augmentation methods.

**Figure 3:**
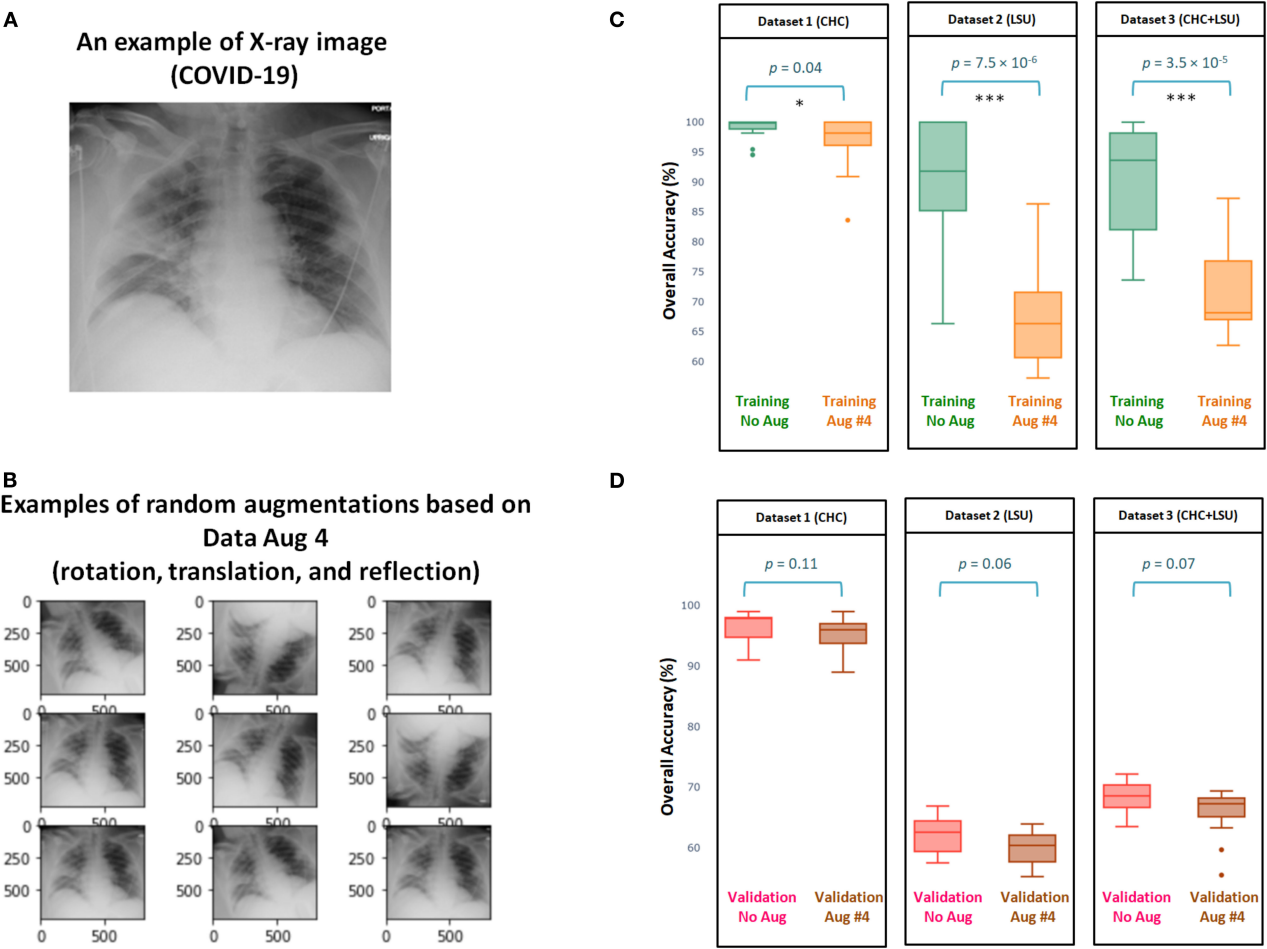
Efficacy of Data Augmentation 4 (6) on X-ray images with and without COVID-19. **(A)** An example of applying Data Augmentation 4, which includes rotation by 10 degree, translation, and horizontally reflection to a COVID-19 X-ray image. **(B)** Comparing the overall accuracy of 17 deep neural networks to examine the efficacy of Data Augmentation 2 (21) when applied to three datasets. **(C)** Boxplots to show the overall statistical different between training with and without augmentation over three datasets. **(D)** Boxplots to show the overall statistical different between validation with and without augmentation. Note that results shown in **(C,D)** are obtained from cross validation with *K* = 10.

Figure 3 shows the efficacy of Data Augmentation 4 on three datasets. Similar to Data Augmentation 2, the augmentation methods help prevent overfitting; however, it fails in generalization as its validation accuracy is less than without augmentation. It is worth mentioning that the overall validation accuracy without augmentation was *not* significantly different from the overall validation accuracy with Augmentation 4, as *p* = 0.11, *p* = 0.06, *p* = 0.07, for Dataset 1, Dataset 2, Dataset 3, respectively (Figure 3D). The performance of Augmentation 4 was ranked first compared to the other augmentation methods based on the MCC, as its average MCC = 0.49 is closest to the MCC without augmentation MCC = 0.51, as shown in Table 1.

**Figure 4:**
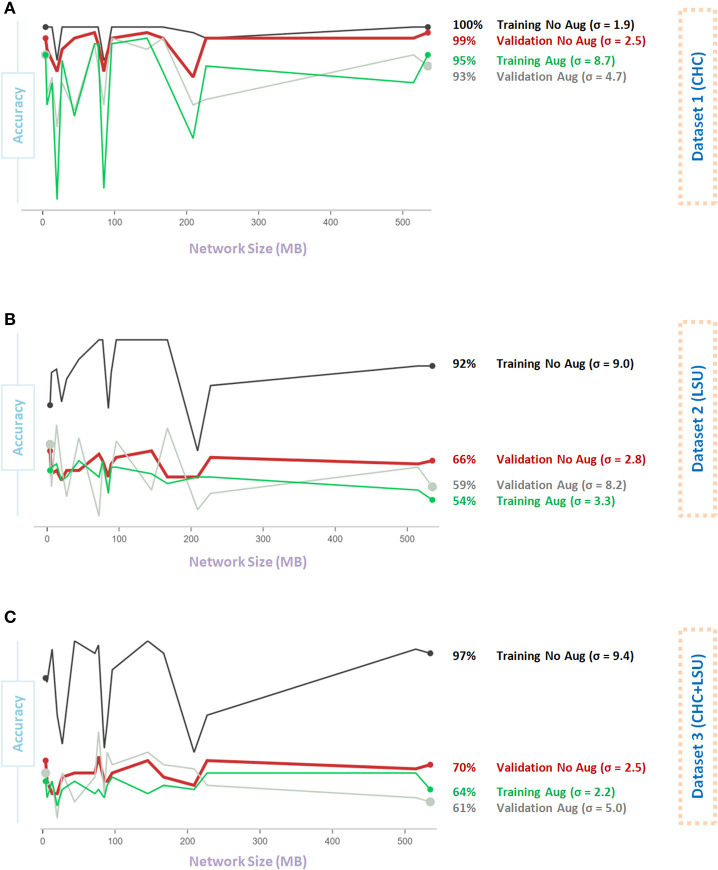
Effect of network size on Data Augmentation 2 (22) for detecting X-ray images with and without COVID-19. **(A)** CHC Dataset. **(B)** LSU Dataset. **(C)** CHC+LSU Dataset. Here, σ refers to standard deviation of performance with respect to network size, which is calculated to measure stability over different network sizes, high value means less stability.

Figure 4 shows the impact of Data Augmentation 2 on network architectures with smaller size, which led to unstable (fluctuating with high variance) performance. As the model size increases from 200 to 500 MB, the generalization accuracy becomes more stable. Small networks, <200 MB, have a limited capacity, which introduces an additional bias that could destabilize generalization, leading to overfitting. Interestingly, over all three datasets, the overall validation accuracy without augmentation is higher than the overall validation accuracy with Data Augmentation 2. In fact, the overall validation accuracy without augmentation was almost stable (σ = 2.5, σ = 2.8, σ = 2.5, for Dataset 1, Dataset 2, and Dataset 3, respectively) and consistent over different network sizes compared to that with augmentation, where σ = 4.7, σ = 8.2, σ = 5.0, for Dataset 1, Dataset 2, and Dataset 3, respectively, as shown in Figures 4A–C.

**Figure 5:**
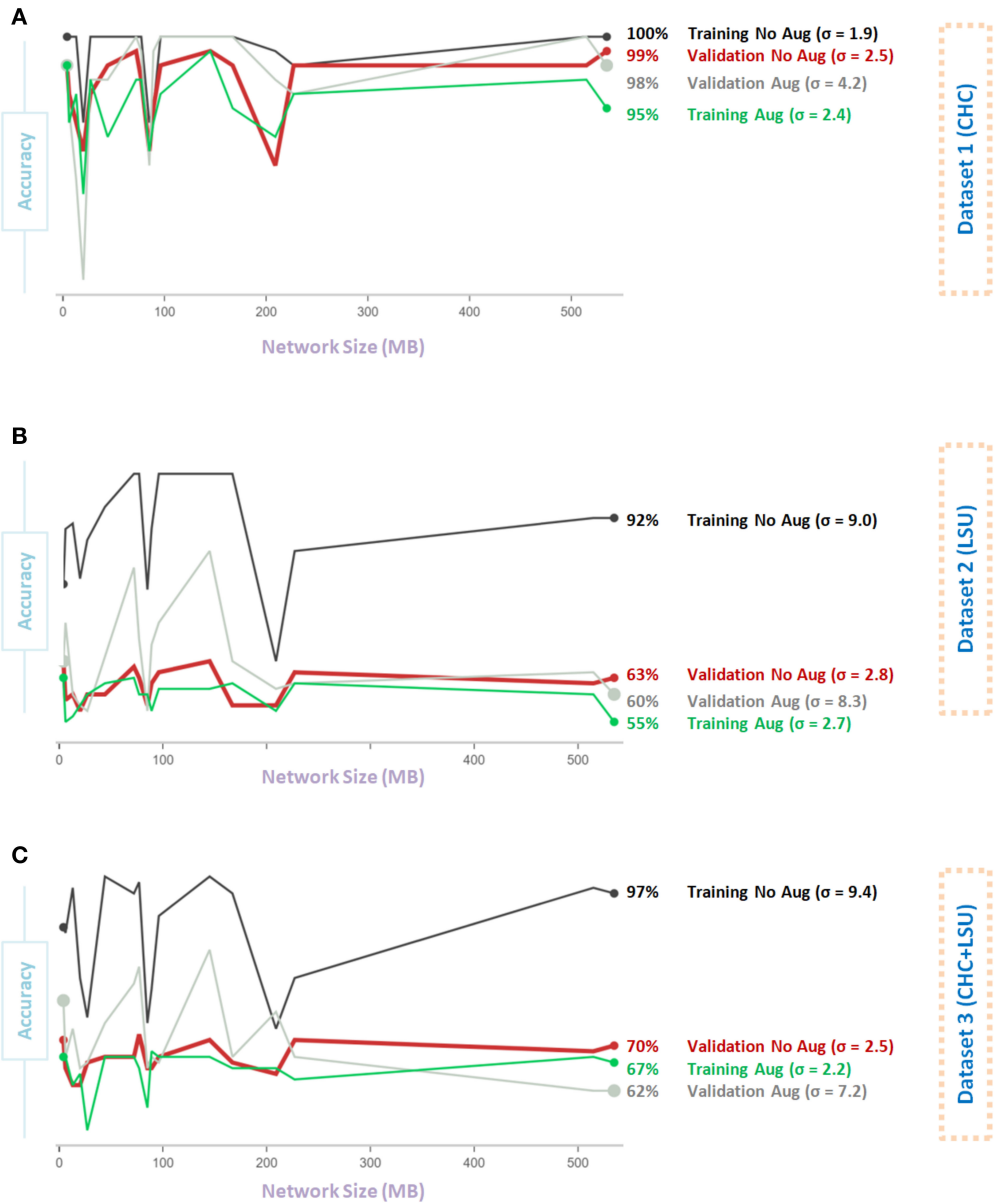
Effect of network size on Data Augmentation 4 (6) for detecting X-ray images with and without COVID-19. **(A)** CHC Dataset. **(B)** LSU Dataset. **(C)** CHC+LSU Dataset. Here, σ refers to standard deviation of performance with respect to network size, which is calculated to measure stability over different network sizes, high value means less stability.

Figure 5A shows that Data Augmentation 4 was relatively stable (σ = 2.5) regardless of the network size over the CHC dataset. Over the CHC, LSU, and CHC+LSU, the larger the network size, the accuracy of models with Data Augmentation 4 could get higher than without augmentation, as shown in Figures 5A–C. However, it is not a stable result, as σ = 4.2 and σ = 8.3 with augmentation compared to σ = 4.2 and σ = 8.3 without the augmentation step on Dataset 1 and Dataset 2, respectively. In other words, one large network size can improve the overall validation accuracy with augmentation 4. However, it does not mean that Data Augmentation 4 will have the same (or similar) effect using a different network. Stability over different sizes is vital to evaluate the impact of the data augmentation method.

A recently published model (19) used an augmentation step that rotated the X-ray images by random angles in the range [−3, 3] degrees and resized the X-ray images by random scale factors in the range [1, 2]. We sought to remove the augmentation step of this algorithm and examine if our finding was valid. We then removed the augmentation step and reran the whole analysis (19) to compare the impact of the data augmentation step.

**Figure 6:**
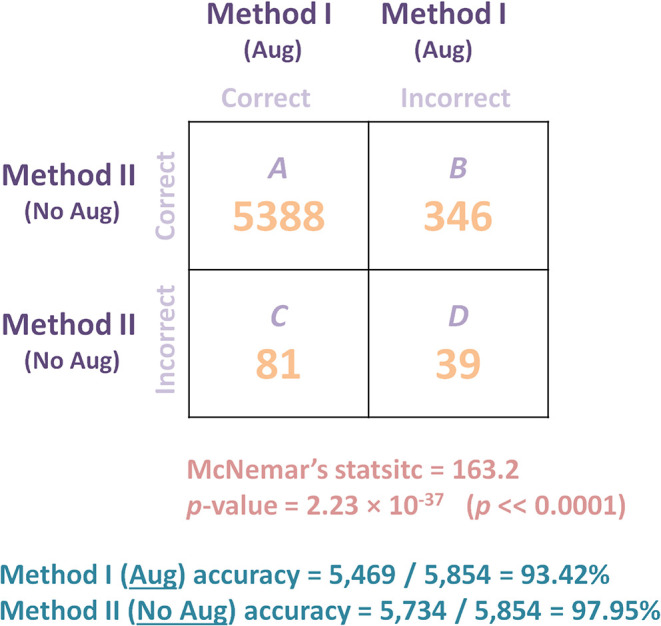
Comparing the performance of two models (with and without augmentation) on Dataset 4 (Testing) of a recently published algorithm (19) for detecting COVID-19 after training the algorithm on Dataset 1 (Training and Validation). McNemar's Test was used to compare the predictive accuracy of two models: Method I (augmentation) and Method II (no augmentation). *B* is the number of X-ray images that were detected correctly by Method II and incorrectly detected by Method I, while *C* is the number of X-ray images that were detected correctly by Method I and incorrectly by Method II. Results show that the deep learning model without augmentation (Method II) significantly outperformed the same model with augmentation.

As shown in Figure 6, Method II resulted in 346 predictions that were correct when compared to Method I did not predict. On the contrary, Method I resulted in 81 correct predictions that Method II did not predict. Thus, based on this 346:81 ratio, we may conclude that Method II performs substantially better than Method I. However, to quantify the impact, the accuracy and the statistical significance need to be reported. The accuracy of each method can be calculated as follow: Method I (Aug) accuracy = 5, 469/5, 854 = 93.42% and Method II (No Aug) accuracy = 5, 734/5, 854 = 97.95%. Based on accuracy calculation, Method II outperformed Method I, suggesting that adding the augmentation step is decreasing the detection accuracy. We then applied the McNemar's Test, and we obtained a $\chi_{\text{McNema}{\text{r}}^{\text{′}}\text{s statistic}}^{2}\;$= 163.2, with a *p*-value of 2.23 × 10^−37^, which is below the set significance threshold (α = 0.05) and lead to the rejection of the null hypothesis; we can conclude that the methods' performances are different. In fact, Method II significantly outperformed Method I. Researchers often blindly assume that applying any data augmentation step improves accuracy, but this is not always the case, and could be dependent on the application and augmented data utilized.

###  Clinical Perspective

Data augmentation is a great tool that can provide new images that preserve original features, but it can also generate noise that can be harmful to the training phase. As an example, applying rotations and flips for detecting a dog in an image, such as in ImageNet challenges (45), is considered “acceptable.” On the contrary, applying the same geometrical augmentations for classifying a digit such as 6 vs. 9, is “not recommended” (5). If the purpose was to recognize a dog in an image, then rotation with a wide range could be acceptable, but if the purpose was to detect COVID-19 in an X-ray image, then rotation could harm the training phase. Furthermore, the accuracy of the deep learning is heavily impacted by the rotation degree.

In computer vision, it seems that applying geometric augmentation steps such as rotation and reflection are generally “acceptable.” However, applying the augmentation step must be sensible and plays an effective role in detecting the required pattern. In other words, the network trained with augmentation needs to be more robust and accurate than expected variations of the same X-Ray images. The augmentation step is a domain dependent, not an arbitrary step, that can be applied to all research fields in the same way.

Applying an augmentation without a clinical consideration may lead to achieving lower accuracy on the validation dataset. Below, we try to discuss different geometric augmentations and label them clinically “acceptable,” “possible,” “not recommended.” This may help organize what to consider and what not to consider during the development of a deep learning algorithm in terms of data augmentation.

After augmentation, the generated augmented images do not get labeled by radiologists to confirm that their validity. Getting radiologists to label original X-ray images is challenging already given their limited time. The requirement to label the augmented images is not practical and perhaps cannot be facilitated.

To close this gap, and speed up the process, between the computer scientists and clinicians, we sought the opinion of radiologists on the different geometric augmentation steps. This could help in designing new algorithmic approaches for detecting COVID-19 using X-ray images in the near future. Presented here is some of that clinical input for consideration:

1. **Reflection**: Reflection in x-axis is a step that is unusual, as the x-ray is flipped upside down. This step is “not recommended,” as it is an adding unnecessary noise that may mislead the learning algorithm. For example, applying this step for digit recognition is “not recommended,” the neural network will not be able differentiate between number 6 and number 9. Reflection in y-axis does *not* change Posterior-Anterior (PA) to Anteroposterior (AP). However, it would lead to non-physiologic images (e.g., heart in the right thorax rather than the left thorax), which might confound learning and is “not recommended.” There is no existing data augmentation technique that can simulate the differences between PA and AP chest x-ray images, since relative positioning in patient, x-ray tube, and detector produce differential magnification and affect edge definition.
2. **Rotation**: Applying rotation to X-ray images could be helpful. However, it depends on the range of rotation a severe rotations can be harmful. Slight rotations such as between −5 and 5 are seen in clinical practice, however, severe rotations such as between −90 and 90 are “not recommended,” as the generated X-rays are unlikely to be encountered, and can add unnecessary noise to the learning model.
3. **Scaling**: Scaling can be in x-axis, y-axis, or both. When large scaling (>× 1) is applied, regardless of the direction, the augmented X-ray image will be a stretched version of the original X-ray. When a small scaling (<× 1) is applied, the size augmented X-ray will be less than the original image. An equal scaling in x-axis and y-axis is “possible,” however, scaling in only the x-axis or y-axis can be considered “not recommended” clinically.
4. **Shearing**: Shearing can be applied to x-axis, y-axis, or both directions. It is measured as an angle in degrees, and is in the range −90 to 90. The augmented X-ray images look like the original skewed in the specified direction(s). This step can be considered “not recommended,” as it produces images that do not exist clinically.
5. **Translation**: Translation or “Shifting” X-ray images up, down, left, or right, could be a useful augmentation step. This is because the X-ray images do not always produce lungs in the center of the image. This can depend on the patient's position, as well as the radiographic unit itself, such as if it is portable. Having X-ray images where the lungs are centered could lead to a more robust COVID-19 detector. As such, this step seems to be “acceptable” clinically as it is observed. However, there is no clearly recommended range for translation.

Scaling augmentation may be a useful method in computer vision applications, especially for capturing a certain pattern in an image. However, for the purpose of detecting COVID-19, it can negatively impact detection accuracy. Unfortunately, applying rotation and scaling augmentations to deep neural networks can reduce the classification accuracy (46). So far, we have tested a subset of data augmentation methods, specifically geometric transformations. There are other augmentation methods that need to be tested, such as color space augmentations, feature space augmentations, and adversarial training.

The main goal of this paper was not to explore the optimal deep learning algorithm to detect COVID-19; rather, the purpose was to examine the impact of simple geometric transformations on the overall performance of detecting COVID from X-ray images. Based on the results obtained by a recent study (19), DarkNet-19 performed better without the augmentation step than with augmentation.

## Limitations and Recommendations for Future Work

There are several limitations to this work. First, we did not investigate all possible combinations of augmentation methods, and only relying upon combinations used in four scientific articles (6, 21–23) published recently. Second, a sample testing dataset of true positives provides an incomplete view of the deep learning model's performance. The overall performance for all datasets (i.e., healthy, non-COVID-19 viral pneumonia, and bacterial pneumonia) before and after data augmentation would show the wider impact of data augmentation. Third, we did not segregate the X-ray images based on technique PA vs. AP nor patient positioning (supine vs. erect versus semi-erect for example), which could have resulted in errors. Although lateral X-rays were excluded, and only AP and PA were used, it is possible that including only one view, such as AP (the most common), might improve the overall accuracy. Finally, we included only non-COVID-19 viral pneumonia and bacterial pneumonia, and including other respiratory pathology might improve the specificity of the deep learning algorithm in detecting COVID-19.

1. We are fortunate that were given access to COVID-19 X-ray images from VGH and LSU. There is the need, however, for a publicly available X-ray dataset that contains a large sample size for COVID-19 collected across a diverse population (e.g., males, females, young, and old).
2. Future research studies need to include the duration between the time of capturing X-ray images and the time of PCR test. Sometimes the X-ray looks clinically normal, however, the PCR reports COVID-19.
3. When developing a COVID-19 detector via X-ray images, it is important to report results with and without augmentation.
4. Optimization over each geometric augmentation is needed. For example, defining an “acceptable” range of rotation.
5. Consistent listing or exclusion of confounders for each X-ray image, such as BMI extermes can help with the development of a robust COVID-19 detector.
6. Future research must consider investigating building a COVID-19 detector for each measurement position PA or AP.
7. There is the need to create a COVID-19 datasets with and without electrodes, tubes and other external radio-opaque artifacts.
8. One of the next steps is to quantify the effect of each geometrical transformation on different datasets. This can be carried out by calculating the average improvement in validation accuracy for each transformation when they are added to a random subset of transformations (47).

## Conclusion

In conclusion, these findings indicate that geometrical data augmentation in X-ray images may not be an effective strategy in detecting COVID-19. Unlike other computer vision learning problems, in which a certain pattern, such as a dog vs. a cat, can be located somewhere in an image, COVID-19, bacterial, and non-COIVD-19 viral pneumonias do not have specific shapes or dimensions on a chest X-ray image. Moreover, the overall validation accuracy without augmentation was almost stable and consistently higher than with augmentation over three datasets. These findings could improve currently published algorithms for the purpose of COVID-19 detection and could guide future research on the topic.

## Data Availability Statement

The original contributions presented in the study are included in the article/supplementary material, further inquiries can be directed to the corresponding author/s.

## Ethics Statement

The studies involving human participants were reviewed and approved by The University of British Columbia. The patients/participants provided their written informed consent to participate in this study.

## Author Contributions

ME designed the study, analyzed data, and led the investigation. MN, DS, J-PG, CB, BS, WL, WP, and SN provided two X-ray datasets, annotated X-ray images, and checked the clinical perspective. ME, MN, QT, DS, J-PG, CB, BS, WL, WP, SN, RF, NH, CM, and RW conceived the study and drafted the manuscript. All authors approved the final manuscript.

## Conflict of Interest

The authors declare that the research was conducted in the absence of any commercial or financial relationships that could be construed as a potential conflict of interest.
